# Enabling Environments? A Spotlight on Community Mental Health Team Offices in Wales

**DOI:** 10.1192/bjo.2025.10613

**Published:** 2025-06-20

**Authors:** Oliver John, Shane Mills, Adrian Clarke, Alka Ahuja, Dafydd Huw

**Affiliations:** 1 Royal College of Psychiatrists Wales, Cardiff, United Kingdom; 2 NHS Wales, Cardiff, United Kingdom

## Abstract

**Aims:** A CMHT office should provide a comfortable, supportive, and therapeutic environment for staff and visitors. It should be accessible and welcoming, it should support the development and maintenance of good relationships, recognition of boundaries and make staff and service users feel physically and emotionally safe.

A CMHT office should enable people to communicate effectively, especially those with differing abilities, cultural differences and languages and it should encourage involvement.

Welsh Government commissioned NHS Wales’ Joint Commissioning Committee and RCPsych Wales to audit all CMHTs in Wales against these principles.

**Methods:** A 109-point specification focused on the environment of care was developed. All points were classed as either ‘desirable’, or ‘essential’, based on legal or regulatory requirements, potential impact on staff safety, effectiveness, or the possible impact on service user safety, outcomes, inclusion or experience.

The specification was split into 10 areas: Build & Maintenance; Enabling Access; External Areas; Internal Areas; Experience, Privacy & Dignity; Equity; Supporting & Protecting Staff; Clinical Care; Health & Social Care Integration; and Community Links.

The specification was designed so the review team could allocate one of three indicative ‘positions’ in response to each question, corresponding to whether a particular aspect of the CMHT office was:

‘Poor/substandard/not present’,

‘Adequate/reasonable/acceptable’ or

‘Good/effective/present’.

A single auditor was used for site visits to support comparative evidence gathering. All Health Boards agreed to participate, and all 45 CMHT offices in Wales were subject to a site visit. During these site visits the environment was assessed, documentation reviewed, and staff interviewed.

**Results:** Across the 109 point specification, there were stark findings.

Examples of ‘more than two-thirds’:

89% of CMHT office external areas were tidy.

89% of CMHT offices were less than 5 minutes walk from a bus stop.

Examples of ‘less than a third’:

24% of CMHT offices had the facility to dispense medications.

22% of CMHT offices parking areas were secure.

Examples of Inequalities in Care:

20% of CMHT offices provided no disabled parking for service user.

2% of CMHT offices had BSL proficient staff and/or access to VSL technology.

**Conclusion:** This audit highlights effective joint working between RCPsych Wales and NHS Wales’ Joint Commissioning Committee. It further highlights that:

Strategic investment is necessary to enhance the CMHT environment in Wales,

Investment must seek to address inequalities in care that are experienced due to the design and state of environments.

